# The association between social capital and mental health and behavioural problems in children and adolescents: an integrative systematic review

**DOI:** 10.1186/2050-7283-2-7

**Published:** 2014-03-26

**Authors:** Kerri E McPherson, Susan Kerr, Elizabeth McGee, Antony Morgan, Francine M Cheater, Jennifer McLean, James Egan

**Affiliations:** Institute for Applied Health Research, School of Health & Life Sciences, Glasgow Caledonian University, Cowcaddens Road, Glasgow, G4 0BA UK; GCU London, 40 Fashion Street, Spitalfields, London, E1 6PX UK; School of Nursing Sciences, Faculty of Medicine and Health, University of East Anglia, Norwich Research Park, Norwich, NR4 7TJ UK; Glasgow Centre for Population Health, 1st Floor, House 6, 94 Elmbank Street, Glasgow, G2 4DL UK

**Keywords:** Family social capital, Community social capital, Children, Adolescents, Mental health, Wellbeing, Behavioural problems, Self-esteem, Internalising behaviours, Externalising behaviours

## Abstract

**Background:**

Mental health is an important component of overall health and wellbeing and crucial for a happy and meaningful life. The prevalence of mental health problems amongst children and adolescent is high; with estimates suggesting 10-20% suffer from mental health problems at any given time. These mental health problems include *internalising* (e.g. depression and social anxiety) and *externalising* behavioural problems (e.g. aggression and anti-social behaviour). Although social capital has been shown to be associated with mental health/behavioural problems in young people, attempts to consolidate the evidence in the form of a review have been limited. This integrative systematic review identified and synthesised international research findings on the role and impact of family and community social capital on mental health/behavioural problems in children and adolescents to provide a consolidated evidence base to inform future research and policy development.

**Methods:**

Nine electronic databases were searched for relevant studies and this was followed by hand searching. Identified literature was screened using review-specific inclusion/exclusion criteria, the data were extracted from the included studies and study quality was assessed. Heterogeneity in study design and outcomes precluded meta-analysis/meta-synthesis, the results are therefore presented in narrative form.

**Results:**

After screening, 55 studies were retained. The majority were cross-sectional surveys and were conducted in North America (n = 33); seven were conducted in the UK. Samples ranged in size from 29 to 98,340. The synthesised results demonstrate that family and community social capital are associated with mental health/behavioural problems in children and adolescents. Positive parent–child relations, extended family support, social support networks, religiosity, neighbourhood and school quality appear to be particularly important.

**Conclusions:**

To date, this is the most comprehensive review of the evidence on the relationships that exist between social capital and mental health/behavioural problems in children and adolescents. It suggests that social capital generated and mobilised at the family and community level can influence mental health/problem behaviour outcomes in young people. In addition, it highlights key gaps in knowledge where future research could further illuminate the mechanisms through which social capital works to influence health and wellbeing and thus inform policy development.

**Electronic supplementary material:**

The online version of this article (doi:10.1186/2050-7283-2-7) contains supplementary material, which is available to authorized users.

## Background

Mental health has been defined by the World Health Organization (2013) as “a state of well-being in which every individual realizes his or her own potential, can cope with the normal stresses of life, can work productively and fruitfully, and is able to make a contribution to her or his community”. However, while mental health is an essential component of general health and wellbeing, mental ill-health is recognised as a significant contributor to the global burden of disease (World Health Organization 2008). Estimates suggest that 10-20% of young people suffer from mental health problems at any given time, with problems being more common during the adolescent years than in childhood (World Health Organization 2003; World Health Organization 2012b). Mental health problems in children and adolescents are important as they are known to influence quality of life, engagement in risky behaviours, behaviour and attendance at school, educational achievement and future health and life chances (Rapport et al. 2001). In addition, pre-adult onset is known to be a major risk factor for mental health problems in later life (Kessler et al. 2005; Kim-Cohen et al. 2003).

Evidently, mental health/ill-health is a multifaceted construct, encompassing a range of positive and negative social, emotional and behavioural dimensions (Thirunavurakasu et al. 2011). While debates continue about what constitutes mental health and wellbeing, mental health problems in adults are generally categorised using the International Classification of Diseases (ICD-10) or the Diagnostic and Statistical Manual of Mental Disorders (DSM-IV) (American Psychiatric Association 2000; World Health Organization 2011). ICD-10 categorises mental health problems into one or more of a number of broad categories including: schizophrenia/schizotypal disorders; affective disorders (e.g. depression); neurotic/stress-related disorders (e.g. anxiety); personality disorders (e.g. psychopathy); disorders of psychological development; and, disorders linked to the use of psychoactive substances (e.g. alcohol) (World Health Organization 2011). Concerns have been raised about the appropriateness of the ICD-10 and DSM-IV diagnostic criteria for children and adolescents, including the risk of the “psychiatrization” of problems in young people (World Health Organization 2003). As a consequence the approach taken to categorise mental health problems in young people commonly avoids the use of ICD-10/DSM-IV criteria, with preference being given to the terms *internalising* behavioural problems (including depression and social anxiety) and *externalising* behavioural problems (including aggression and anti-social behaviour) (Achenbach 1992, Almedom 2005, Xue et al. 2005).

Awareness of the potential sustained and long term consequences of mental health problems has, in recent years, resulted in a paradigmatic shift in public/mental health approaches from curative to preventive, particularly in the context of children and adolescents (National Mental Health Development Unit 2013*;* World Health Organization 2002). The focus of national and international policymakers is therefore on creating and supporting opportunities for young people to accumulate and exploit factors, or assets, known to protect and improve mental health and behavioural outcomes (National Institute for Health and Clinical Excellence 2012; World Health Organization 2002). In this regard, a recent review of the literature that explored ways in which children and adolescents construct and experience mental health highlighted a range of risk and protective factors (Shucksmith et al. 2009). Many of the factors highlighted by the young people as being important for their mental health represent constructs that have been described elsewhere as being indicators of ‘social capital’, including, for example, the pivotal role of family and peer relationships and the impact of neighbourhoods and communities (Ferguson 2005, Kawachi et al. 1997; Morgan 2011; Vyncke et al. 2013).

Similar to mental health, social capital is a multifarious construct that has emerged from the works of Pierre Bourdieu (1986), James Coleman (1988) and Robert Putnam (1995). Reflecting their disciplinary backgrounds, each of these theorists has conceptualised social capital differently and this has generated debate in the literature about how social capital should be defined and measured. Bourdieu defines social capital in terms of networks and connections between individuals that can provide support and resource, Coleman conceptualises social capital as being a resource of the social relations that exist between families and the communities that they are linked to, and Putnam defines social capital as a characteristic of communities including community cohesion, reciprocity and trust. While a more nuanced debate about how social capital should be conceptualised continues, theorists such as Kawachi have sought a more pluralistic approach that attempts to unify key elements that emerge from the various traditions. This has resulted in relative consensus that social capital includes those elements of social networks that can bring about positive social, economic and health development (Kawachi et al. 1999; Morgan 2011; Ottebjer 2005) and this can occur at the micro (individual, family/household) and macro (local, national and international) level (Almedom 2005; Morgan 2011; Ottebjer 2005).

Despite, or perhaps because of, its complex nature, social capital has been discussed and debated in the public health field by those wishing to explain, reduce and prevent health inequalities (e.g. Almedom 2005; Carlson and Chamberlain 2003; Gillies 1998; Kawachi et al. 1997; National Mental Health Development Unit 2013). Specific consideration has also been given, by some, to the ways in which social capital might be a resource for the health and wellbeing of young people (Morgan 2010; Morgan 2011; Morrow 1999; Putnam 1995). While an initial paucity of primary research was a constraint (Almedom 2005), the empirical evidence base has accumulated over the past 10 years with a number of studies suggesting that social capital is an important asset for the health and wellbeing of children and adolescents, including for their mental health (Caughy et al. 2008; Drukker et al. 2003; Morgan 2010; Morgan and Haglund 2009; Morrow 2004).

However, it is important to recognise that social capital is a construct developed within an adult framework and, therefore, traditional definitions may be inadequate for children and adolescents (Morgan 2011; Morrow 2001). Young people may differ from adults in terms of the social spaces they inhabit and social connections that they develop and exploit (Morgan 2011; Morrow 2001). Also, children and young people are acknowledged as having agency and autonomy in the health process and capable of generating and using their own social capital (James and Prout 1997). As an example, schools are rarely included in traditional definitions of social capital but they are an important community arena for young people and represent places where family and community intersect and where young people’s social networks can be developed and exploited (e.g. Vieno et al. 2005).

Thus, while research suggests that social capital may offer an appropriate underpinning for interventions designed to promote better mental health and prevent behavioural problems, there have been few attempts to progress a more theoretical approach to understanding social capital, particularly in the context of young people. This limits our ability to develop appropriate and theoretically-driven interventions. Recognising this, Morgan and Haglund (2012) made a recent plea for research designed to support future hypothesis generation and this includes systematic reviews of existing literature that has focused specifically on young people.

A small number of systematic reviews do exist but their contribution to the field is limited. For example, Ferguson undertook a review of the literature to explore conceptual and operational definitions of social capital when used as a predictor variable in the context of children’s wellbeing (Ferguson 2006); however, the definition of ‘wellbeing’ was very broad and does not enable conclusions to be drawn on the association between social capital and mental health and behavioural problems. That said, this review highlights the importance of exploring social capital at both the family and community level when the focus is young people. Additional reviews have been undertaken that have focused more specifically on the influence of social capital on mental health (i.e. Vyncke et al. 2013, Almedom 2005 and De Silva et al. 2005). While adding important information to the evidence base, none of these reviews focused specifically on the mental health/problem behaviours of children and adolescents. Vyncke and colleagues had a very broad definition of health and wellbeing that extended beyond mental health/behavioural problems and Almedon and De Silva et al. focused mainly on adults, making it difficult to draw firm conclusions about young people.

In light of limited review-level evidence this current systematic review took up Morgan and Haglund’s challenge by seeking to: a) identify, analyse and synthesise primary evidence on the association between social capital and mental health and behavioural problems in children and adolescents: and, b) make recommendations/discuss implications for future research and policy development. To the best of our knowledge, this is the first attempt to focus solely on the evidence base in this area. To ensure the review was comprehensive and not limited to one particular theoretical paradigm, we adopted a pluralistic approach and included evidence from across the range of theoretical traditions within the social capital literature. However, with the focus on children and adolescents, and reflecting on the findings of Ferguson (Ferguson 2006) and Almedom (Almedom 2005), we used the concepts of family social capital (FSC) and community social capital (CSC) (see the *Types of social capital* section below for more detail on the elements of FSC and CSC) as a framework to guide the extraction and synthesis of the data and to structure the presentation of the results.

## Methods

This systematic review was part of a larger review that explored the association between social capital and a broad range of psychosocial health and wellbeing in children and adolescents. In the larger review health and wellbeing outcomes were grouped in a way that would offer the greatest conceptual and practical value (e.g. mental health and behavioural problems, health risk behaviours, health promoting behaviours). In this paper, mental health and behavioural problem outcomes were grouped together and analysed and reported on distinctly from other health and wellbeing domains which have been reported elsewhere (e.g. McPherson et al. 2013b).

Given that the purpose was to synthesise existing empirical research to provide a consolidated overview of the evidence in this field of study, rather than the generation of new theory, we adopted an integrative approach which enables the synthesis of different types of evidence (i.e. qualitative, quantitative and mixed-methods) (Dixon-Wood et al. 2005). In the larger review (n = 102 papers) we employed a single search strategy to identify relevant literature across the range of health and wellbeing outcomes and this search strategy is available as part of the full method in the final report (McPherson et al. 2013a). Here we present the elements of the method directly relevant to the identification and synthesis of data on mental health and behavioural problems (i.e. internalising and externalising behavioural problems). A copy of the larger review protocol is available on request from the lead author.

### Criteria for inclusion

#### Types of studies

Facilitated by our integrative approach we sought to include primary empirical quantitative, qualitative and mixed methods studies that were published and peer reviewed.

#### Types of participants

Studies were included if they focused on preschool children, school-aged children and/or adolescents. Scoping of the literature revealed inconsistencies in the ways that authors defined children and adolescents, we therefore adopted a pragmatic approach, guided by the WHO’s definition of adolescence (World Health Organization 2013). Samples where the majority were 10-19 years old were described as ‘adolescents’, samples where the majority were 5-10 years old were described as ‘children’, and samples where the majority of children were 0-5 years old were described as ‘preschool children’. We also included ‘mixed age group’ samples.

We included studies where the data had been collected directly from the young person and where the data about the young person had been reported by a relevant other (e.g. parent, teacher or professional).

#### Types of social capital

While we took a pluralistic approach to the conceptualisation of social capital, we drew on Ferguson’s (Ferguson 2006) findings as a framework for categorising indicators of family and community social capital. Therefore, only studies that included an indicator of family and/or community social capital were considered for inclusion. The elements of family social capital (FSC) included: family structure (e.g. number of parents present in the household); the quality of parent–child relations (e.g. parent–child communication); adult interest in the child (e.g. parental involvement with school); parent’s monitoring of the child (e.g. perceptions of parental monitoring/control); and, extended family support and exchange (e.g. perceptions of extended family support). The elements of community social capital (CSC) included: social support networks (e.g. peer support); civic engagement in local institutions (e.g. volunteering); trust and safety (e.g. trust in others); religiosity (e.g. attendance at religious services); the quality of the school (e.g. school cohesion and relationship between teachers and pupils); and, the quality of neighbourhood (e.g. neighbourhood cohesion and social control). We also considered studies that employed a composite measure of family and/or community social capital and studies where, although the indicator did not fit within the definition above, the author(s) explicitly described their work as family (e.g. family cohesion) and/or community social capital (e.g. adult role models) and we refer to this as ‘other measure’. Only studies that conceptualised and/or measured social capital as predicting, or influencing, mental health and/or behavioural problems were considered for inclusion; studies were not included if they conceptualised social capital as an outcome variable.

#### Types of outcomes

Studies were included if they assessed individual-level mental health and/or behavioural problems. The outcomes considered included: self-esteem and self-worth; internalising behaviours which includes thoughts, feelings, emotions and behaviours that the child/adolescent directs inwards (e.g. depression and anxiety); and, externalising behaviours which includes the outward expression of feeling and emotions (e.g. aggression, violence, conduct disorders and disobedience). We also considered studies where researchers had measured internalising and externalising behaviours on a single scale giving a composite assessment of mental health and behavioural problems. Only studies that conceptualised and/or measured mental health and/or behavioural problems as outcome variables were considered for inclusion.

### Search strategy

#### Data sources

Nine electronic bibliographic databases were searched in April 2012 including: ASSIA, CINAHL, Cochrane Database of Systematic Reviews, Cochrane Central Register of Controlled Trials, Database of Abstracts of Reviews Effects, Embase, Medline, PsycINFO, and Sociological Abstracts. We also hand searched the reference lists of retrieved articles and web-sites of organisations and groups conducting research on the health and wellbeing of children and adolescents, and/or research in the field of social capital. The websites included: the Centre for Research on Families and Relationships, the World Health Organization and the Social Capital Task Group (Edinburgh, UK).

#### Search terms and delimiters

To identify appropriate search terms, we undertook initial scoping of relevant electronic databases. As noted above, this review was part of a larger piece of work exploring the association between FSC and CSC and children and adolescents’ individual-level psychosocial health and wellbeing outcomes and we developed a single strategy to capture literature from across the range of outcomes, including mental health and behavioural problem outcomes. The search strategy included both index terms (i.e. thesaurus and subject headings) and free text keywords and combined social capital-relevant search terms (e.g. family social capital, community social capital and social networks) with health and wellbeing outcome-relevant search terms (e.g. mental health, emotional adjustment and behaviour problems). The search strategy was appropriately tailored for each database (Evans 2002) and the PsycINFO search strategy is presented in Additional file 1.

We limited our searches to literature published between January 1990 and April 2012 and to English language-only. Retrieved articles were stored in *RefWorks*.

### Data collection and analysis

#### Selection of studies

Duplicates were removed and identified articles were subject to a two-stage screening process. The title and abstract of each article was screened independently by two members of the research team and articles that did not fit the inclusion criteria were rejected. Where no abstract was available the article was retained to the next stage which involved screening of the full text against the inclusion/exclusion criteria; again, this was done by two members of the research team who worked independently of each other.

#### Data extraction

We developed a review-specific data extraction tool to enable the extraction of data from studies with a range of different research designs. Key elements of the extraction included: the context of the study, such as the geographical location and the year(s) of data collection; the aims and purpose of the study; methodological considerations, such as design, participants and data collection methods; the main findings; and, the strengths and limitations of the study. Two reviewers extracted the data from each study independently and any disagreements were resolved through discussion, involving a third reviewer if necessary.

#### Quality appraisal

Quality appraisal was carried out at the same time as data extraction. The two reviewers used a study-specific quality appraisal tool (QAT), which was developed to enable appraisal of studies with a range of research designs. The QAT included 11 criteria covering: whether the theoretical framework underpinning the research was explicitly described; explicit reporting of the study aims and objectives; the concordance between the stated aims and the methodological approach; the rigour and reporting of the results; and, the appropriateness of the conclusions drawn. Criteria were scored on a three-point scale (0 = weak, 1 = moderate, 2 = strong), giving a possible range of scores from 0 to 22 for each study. Disagreements were resolved through discussion, involving a third reviewer if necessary.

Each study was then awarded a quality rating: studies scoring between 16 and 22 were awarded a ‘high quality’ rating; studies scoring between 8 and 15 were awarded a ‘moderate quality’ rating; and, studies scoring between zero and seven were awarded a ‘low quality’ rating. We did not exclude studies on the grounds of quality but the quality scores are presented to facilitate the reader’s interpretation of the findings.

#### Data analysis and synthesis

The majority of the included studies were surveys and there was considerable heterogeneity in the outcome measures employed which prohibited meta-analysis. The low number of identified qualitative studies (n = 1) also prevented us conducting a meta-synthesis. The results are, therefore, presented in narrative form.

Results were summarised and then synthesised using an adaptation of the approach originally described by Ramirez et al. (Ramirez et al. 1999). Specifically, the results were grouped into three categories of association: results that showed a positive association between social capital and mental health and/or behavioural problem outcomes (i.e. where social capital was associated with better outcomes and the results were statistically significant); results that showed a negative association between social capital and mental health and/or behavioural problem outcomes (i.e. where social capital was associated with poorer outcomes and the results were statistically significant); and results where no association between social capital and mental health and/or behavioural problem outcomes was identified (i.e. results were not statistically significant).

Many of the studies included in this review reported on multiple associations between the various elements of family and/or community social capital and various mental health and/or behavioural problem outcomes. Each investigated association is reported in its own right and, therefore, there are many more reported associations between the various elements of social capital and the outcomes than there are included studies.

## Results

### Study selection

**Figure 1:**
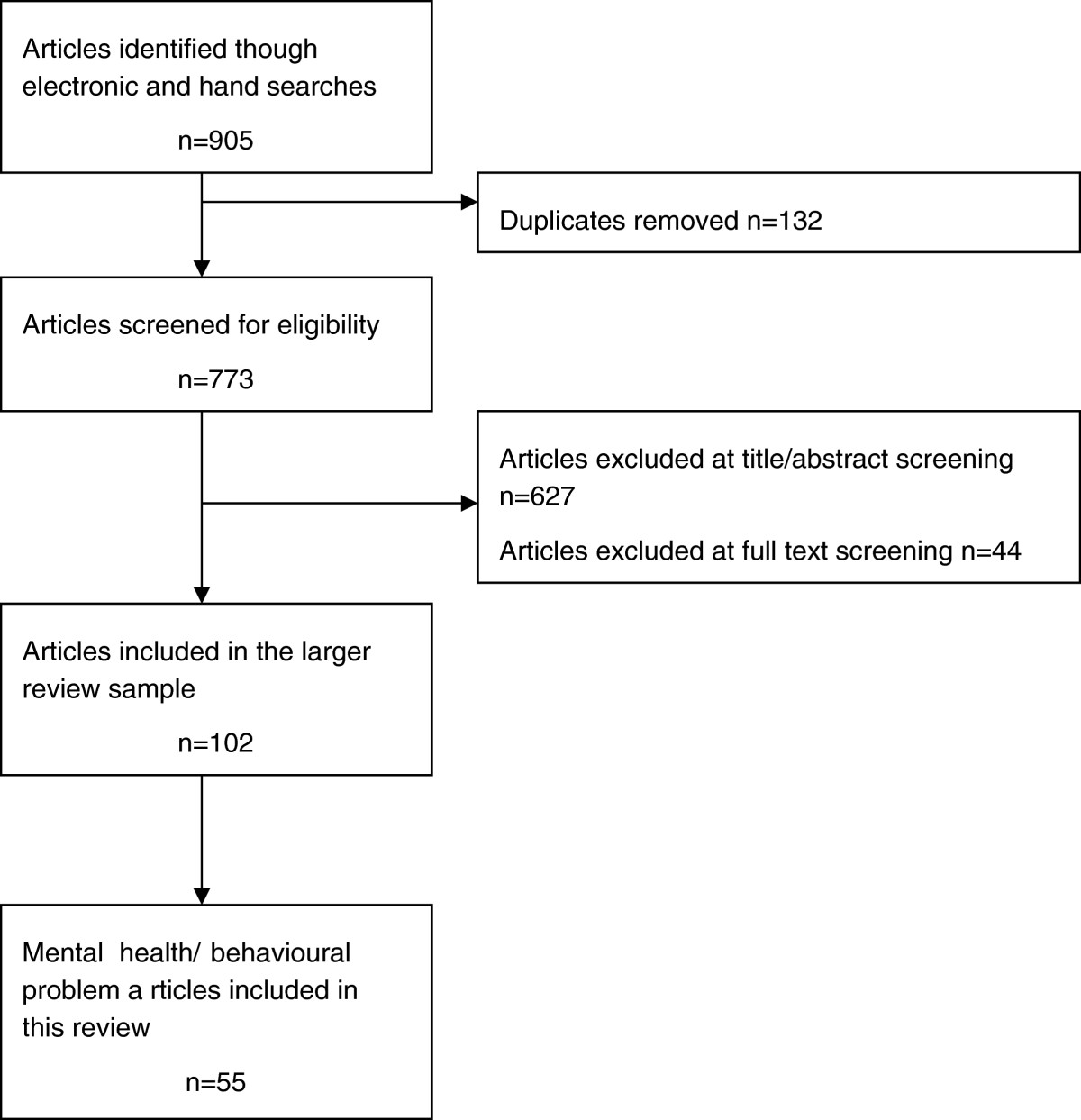
**Flow diagram of search results.**

Following PRISMA (Preferred Reporting Items for Systematic Reviews and Meta-analyses) guidelines (Moher et al. 2009), the search and screening phases are represented in Figure 1. After removing the duplicates, the search yielded 773 unique studies which were screened using the inclusion/exclusion criteria. The majority (n = 627) of the studies were excluded at the title and abstract screening stage and a further 44 were excluded when the full text was screened. Studies were excluded because they did not fit with our definition of child/adolescent (n = 389) or our definition of health and wellbeing (n = 115), the study design criteria (n = 92) or the definition of family or community social capital (n = 73). A total of 102 articles were retained for inclusion across the health and wellbeing outcomes of the larger review; 55 of these included mental health and/or behavioural problem outcomes and represent the total sample reported here.

### Description of studies

The quality appraisal ratings and key descriptive information for each of the 55 included studies is presented in Additional file 2: Table S1. The reviewers rated 37 of the studies as high quality, 17 as moderate quality and one study was assessed as being low quality.

Following data extraction the mental health and behavioural problem outcomes were grouped into four coherent categories: self-esteem and self-worth; internalising behaviours (e.g. depression and anxiety); externalising behaviours, (e.g. aggression, violence, conduct disorders and disobedience); and, composite measures of mental health and problem behaviours. Nineteen studies reported on two or more of these categories. Nine studies reported on at least one indicator of FSC, 22 reported on at least one indicator of CSC and 24 reported on both FSC and CSC (see Additional file 2: Table S1).

The majority of the studies (n = 43) were surveys, and six of these were longitudinal. Also included were eight longitudinal (Birndorf et al. 2005; Drukker et al. 2006; Drukker et al. 2010; Feldman 2010; Parcel and Menaghan 1993; Parcel and Menaghan 1994; Windle 1994; Xue et al. 2005) and one cross-sectional cohort studies (Drukker et al. 2003), one controlled trial (DuBois et al. 2002), a quasi-experiment (Bowker et al. 2010) and one qualitative study (Landstedt et al. 2009). The majority of the studies were conducted in the USA (n = 29), seven were conducted in the UK, four in Canada and four in the Netherlands. The remaining single country studies were conducted in Australia, El Salvador, Greece, Israel, Italy, Lebanon, Serbia, Sweden, Taiwan and Vietnam and one study was conducted in the UK and Canada (see Additional file 2: Table S1).

Few of the studies clearly articulated the dropout rates and in many the number of participants fluctuated across the different analyses that were conducted. To ensure consistency, in this review we report the maximum number of young people included in each study’s analyses. Samples ranged in size from 98,340 participants to 31 in the quantitative studies and in the qualitative study the sample size was 29. Two studies reported on the mental health and/or behavioural problems of preschool children, five reported on children, 34 reported on adolescents and the remaining 14 studies reported on mental health and/or problem behaviour outcomes of mixed aged groups of young people. Thirty-nine studies had mixed sex samples with the percentage of female participants ranging from 45% to 63%. One study had a male-only sample and in the remaining 15 studies, the sex of participants was unclear or not reported.

In 29 studies the ethnicity, race or nationality of the young people was not reported or it was not possible to extract this information. Nine studies described the majority participant group as Caucasian, non-Hispanic White or White and we grouped these under the single category ‘White’ for reporting. Eleven studies described the majority group as African American, Black, or non-Hispanic Black and we grouped these under the single category ‘Black’. In the remaining studies the majority participant groups were described as American Indian, Dutch, Latino, Mainland Chinese and Southeast Asian American.

### Self-esteem and self-worth

Ten studies (see Additional file 2: Table S1) explored the role and impact of social capital on self-esteem or self-worth (Abbotts et al. 2004; Birndorf et al. 2005; Ciairano et al. 2007; Drukker et al. 2006; DuBois et al. 2002; El-Dardiry et al. 2012; Glendinning and West 2007; Jager 2011; Ying and Han 2008; Yugo and Davidson 2007). Four studies, all with adolescent samples, explored the role of FSC and there was evidence that parent-adolescent relationships characterised by positive communication (Birndorf et al. 2005), nurturance (Yugo and Davidson 2007) and low levels of conflict (Ying and Han 2008) were associated with higher self-esteem/worth. Moreover, there was longitudinal evidence showing that positive parent-adolescent relationships in early adolescence were associated with better self-esteem at age 17-18 years (Birndorf et al. 2005). Families assessed as being cohesive (Ying and Han 2008) and families where there was evidence of adult interest in the adolescent (Ying and Han 2008) were associated with better outcomes. This gives further support to the positive role of intra-familial relationships. In contrast, parental monitoring and control was associated with poorer self-esteem/worth (Glendinning and West 2007; Yugo and Davidson 2007).

The seven studies that assessed the role and impact of CSC offered evidence to show that positive relationships that extend beyond the family boundaries are associated with higher levels of self-esteem/self-worth. Children and adolescents were more likely to report higher self-worth/esteem if they had access to their own support networks that include both adults (DuBois et al. 2002) and their peers (Glendinning and West 2007; Yugo and Davidson 2007). They also benefited from their parents’ networks, with better outcomes being reported in children/adolescents whose parent(s) received support from informal networks/experienced a sense of belonging and support (El-Dardiry et al. 2012).

The quality of the school the adolescent was attending was associated with positive self-esteem/worth. Adolescents who reported feeling safe at school (Birndorf et al. 2005) and adolescents who reported that they were engaged with school (Yugo and Davidson 2007) had more positive self-esteem/self-worth. However, one study that explored school quality in sub-groups of adolescents reported it to be associated with self-esteem/worth in adolescents from urban communities but not those from rural communities (Glendinning and West 2007).

There was also evidence that religiosity had a differential impact across sub-groups of adolescents. Increased attendance at religious services was associated with better outcomes for male adolescents (Birndorf et al. 2005) and weekly attendance at religious services was associated with better outcomes for adolescents who self-identified as Catholic; however, church attendance was associated with poorer outcomes in adolescents self-identifying as belonging to the Church of Scotland (Protestant) (Abbotts et al. 2004). There was no data available to explore this further in this review, but the authors hypothesise that differences in the normative behaviours of religious groups may play a role here (e.g. church attendance may be more accepted in some groups than others).

In sum, adolescents who share a positive relationship with their parent(s) and those with higher quality/quantity of social support networks are more likely to have higher self-esteem/worth. On the other hand, parental monitoring/control, which may reflect more negative elements of the parent-adolescent relationship, appears to be linked with lower self-esteem/worth perhaps reflecting the adolescents’ loss of autonomy in aspects of their own lives.

### Internalising behaviours

Thirty-one studies explored the role and impact of social capital on internalising behaviours (see Additional file 2: Table S1). The specific outcomes in these studies include: depressive symptoms, anxiety and social anxiety, moods, emotions and composite scores on assessments that measure a range of these behaviours (referred to by some authors as ‘over-controlled behaviours’) (Abbotts et al. 2004; Aneshensel and Sucoff 1996; Beiser et al. 2011; Bosacki et al. 2007; Caughy et al. 2003; Caughy et al. 2006; Caughy et al. 2008; Ciairano et al. 2007; Delsing et al. 2005; Drukker et al. 2003; Drukker et al. 2006; DuBois et al. 2002; El-Dardiry et al. 2012; Fitzpatrick et al. 2005; Fulkerson et al. 2006; Glendinning and West 2007; Jager 2011; Kliewer et al. 2004; Landstedt et al. 2009; Meltzer et al. 2007; Rasic et al. 2011; Rotenberg et al. 2004; Rotenberg et al. 2005; Springer et al. 2006; Stevenson 1998; Wang et al. 2011; Windle 1994; Xue et al. 2005; Ying and Han 2008; Young et al. 2011). We also included studies that reported on suicide/suicidal ideation and self-harm.

Although explored by two studies, there was no evidence to suggest that family structure shared an association with internalising behaviours (Aneshensel and Sucoff 1996; Glendinning and West 2007). On the other hand, seven studies presented evidence to suggest that positive parent–child relationships were associated with decreased levels of internalising behaviours in children and adolescents (Caughy et al. 2008; Springer et al. 2006; Ying and Han 2008). Moreover, there was evidence that the quality of the parent–child relationship may be more important for some sub-groups of young people than others. Positive relationships were associated with better outcomes in children/adolescents living in low violence neighbourhoods (Kliewer et al. 2004), a pattern not replicated in high violence neighbourhoods, and adolescents from rural communities benefited from good relations with their parents in a way not afforded to adolescents from urban communities (Glendinning and West 2007).

Further supporting the positive association between family relationships and internalising behaviour outcomes, children and adolescents who assessed their relationships with other family members as high in justice (i.e. fairness) and trust (Delsing et al. 2005), those who were part of a cohesive family (Ying and Han 2008) and those from families that frequently had meals together (Fulkerson et al. 2006) had better internalising behaviour outcomes. In contrast, reports of parental monitoring in two separate studies were inconsistent; one reported a positive association with adolescents’ internalising behaviours (Ying and Han 2008) and the other reported a negative association (Glendinning and West 2007).

Eleven of the included studies explored the role of support networks. There was evidence to suggest that children and adolescents with access to wider social networks (i.e. a higher number of friendships) (Rotenberg et al. 2004) and higher quality social networks (e.g. friendships low in hostility) (Beiser et al. 2011; Windle 1994) had fewer internalising behaviours than children/adolescent with smaller or poorer quality social networks. Again, some sub-groups of children/adolescents may benefit more from social support networks than others. For example, preschool children living in affluent neighbourhoods had fewer reported internalising behaviours if their primary caregiver reported knowing their neighbours, on the other hand, in impoverished neighbourhoods not knowing neighbours was associated with better outcomes for preschool children (Caughy et al. 2003). Moreover, peer support was associated with fewer internalising behaviours in adolescents from rural communities but this was not replicated in urban communities (Glendinning and West 2007).

There was evidence to suggest that schools and neighbourhoods with higher quality environments offered children and adolescents protection in relation to internalising behaviours. Cohesive neighbourhoods (Kliewer et al. 2004), neighbourhoods low in hazards (Aneshensel and Sucoff 1996) and neighbourhoods high in other indicators of social capital were associated with lower internalising behaviours. Only one study reported a negative association between neighbourhood quality and internalising behaviours; adolescents who perceived that adults in their neighbourhood imposed too many constraints on them reported higher levels of internalising behaviour (Glendinning and West 2007). Although the authors did not explore this further, it might be hypothesised that while control over adolescent behaviour (e.g. anti-social behaviour) may improve the quality of the neighbourhood in the eyes of adult residents this may not be perceived as such by adolescent residents.

In sum, children and adolescents with more positive relationships with other family members and who have wider and higher quality networks that extend beyond the family, either directly with their peers or indirectly through their parents’ networks, have fewer reported internalising behaviours. Living in a higher quality neighbourhood is also associated with better child and adolescent mental health outcomes. That said, it is important to note that social support networks may not benefit all children equally. For example, in impoverished communities, better outcomes are reported for children whose primary caregiver reported knowing fewer of their neighbours. The authors hypothesise that mothers who are able to manage adversities in their impoverished neighbourhood, perhaps because they can access other assets, may need less social capital to support healthy development in their children (Caughy et al. 2003).

### Externalising behaviours

Twenty-four studies (see Additional file 2: Table S1) explored the role and impact of social capital on externalising behaviours and these included measures of: aggression; anger; violence; lying; conduct and oppositional defiant disorder symptoms (negative, short tempered, defiant, argumentative, disobedient and hostile behaviour towards adults and authority figures); and composite scores on assessments that measure a range of externalising behaviours (referred to by some authors as ‘under-controlled behaviours’) (Abbotts et al. 2004; Aneshensel and Sucoff 1996; Bearinger et al. 2005; Caughy et al. 2003; Caughy et al. 2006; Caughy et al. 2008; Champion et al. 2008; Ciairano et al. 2007; Delsing et al. 2005; Drukker et al. 2003; Drukker et al. 2010; DuBois et al. 2002; El Hajj et al. 2011; Fulkerson et al. 2006; Jager 2011; Johnson 1999; Kingston et al. 2009; Kliewer et al. 2004; Meltzer et al. 2007; Oman et al. 2005; Smith and Barker 2009; Springer et al. 2006; Stevenson 1997; Windle 1994). The evidence available to assess the role and impact of family structure on externalising behaviours was limited to two studies and only one of these found an association; living in a one-parent household was predictive of increased oppositional defiant disorder symptoms (Aneshensel and Sucoff 1996). There was, however, evidence to demonstrate that positive relationships between parents and their adolescent/child were associated with reporting of fewer externalising behaviours (Caughy et al. 2008; Kliewer et al. 2004; Springer et al. 2006). Moreover, the parent-adolescent relationship appears to be particularly important for those from a one-parent household (Oman et al. 2005).

Given the nature of these behaviours, it is perhaps surprising to note that only one of the included studies explored the association between parental monitoring and externalising behaviours and failed to find one (Smith and Barker 2009). However, there was evidence demonstrating that positive relationships between children/adolescents and their extended family were associated with better outcomes. Children and adolescents from families that were high in feelings of trust and justice (Delsing et al. 2005) and cohesion (e.g. more frequently ate meals together) (Fulkerson et al. 2006) had lower levels of externalising behaviours. In contrast, adolescents living in high risk neighbourhoods reported increased suppression of anger when extended family support was higher. The authors suggest this demonstrates that the family has an important role to play in moulding anger suppression in adolescents and, we surmise, that this may be context-specific (Stevenson 1997). Families in neighbourhoods where the risk of violence and/or conflict is high are likely to transmit different messages to young people about appropriate behaviours than families living in neighbourhoods where the risk is low.

There was mixed evidence relating to the association between social support networks and externalising behaviours. In one study, preschool children living in areas with high levels of poverty were reported to be at increased risk of displaying externalising behaviours if their primary caregiver reported higher levels of social support from their neighbours. In contrast, preschool children from more affluent areas were less likely to display externalising behaviours if their primary caregiver reported having social support from neighbours (Caughy et al. 2003). For adolescents, increased quantity and quality of social networks was associated with increased lying and disobedient behaviours in one study (Ciairano et al. 2007) and increased reporting of fighting in another (El Hajj et al. 2011). However, a number of other studies reported that social support networks offered adolescents protection against some externalising behaviours (e.g. fighting, delinquency and anti-social behaviours) (Champion et al. 2008; Oman et al. 2005; Windle 1994).

Also associated with externalising behaviour outcomes was the quality of a child/adolescent’s school and neighbourhood environment. Children and adolescents who attend a higher quality school and/or live in higher quality neighbourhoods are less likely to display externalising behaviours (Aneshensel et al. 1996; Bearinger et al. 2005; Springer et al. 2006).

In sum, in the context of externalising behaviours FSC offers the most consistent protective role for children and adolescents. In the context of CSC a number of studies reported risk relationships and in other studies social capital was protective for some externalising behaviours but not others. Consistent with internalising behaviours, caregivers from impoverished neighbourhoods who reported knowing few of their neighbours also reported better outcomes for their children. As noted above, this may be because these caregivers have access to assets other than social capital that enable them to deal with the demands of their environment and support healthier development in their children (Caughy et al. 2003).

### Composite internalising and externalising behaviours

Thirteen of the included studies (Dorsey and Forehand 2003; Dufur et al. 2008; Feldman 2010; Galboda-Liyanage et al. 2003; Harpham et al. 2006; Maynard and Harding 2010; Maynard and Harding 2010; Newman 2007; Parcel and Menaghan 1993; Parcel and Menaghan 1994; Parcel and Dufur 2001; Slee and Murray-Harvey 2007; Wen 2008) (see Additional file 2: Table S1) explored the role and impact of social capital on internalising and externalising problem behaviours measured as a single outcome on a scale such as the difficulties sub-scale of the Strengths and Difficulties Questionnaire (Goodman 1997). Nine of the studies were cross-sectional surveys and they reported on mixed sex samples across the various age groups.

Family structure was assessed by five studies and the evidence suggested that young people who lived in a two-parent family were less likely to have internalising/externalising problems (Galboda-Liyanage et al. 2003, Wen 2008). There was stronger evidence, in six studies, that positive parent–child relationships were protective against internalising/externalising problems in children and adolescents (Feldman 2010; Maynard and Harding 2010; Parcel and Dufur 2001; Wen 2008). There was inconsistent evidence for the role of parental monitoring with one study reporting a negative impact of control for adolescents (Maynard and Harding 2010) and another reporting monitoring to be positive for children and adolescents (Parcel and Dufur 2001). Total FSC, assessed using a composite measure, was also associated with better child/adolescent outcomes (Dorsey and Forehand 2003; Dufur et al. 2008).

Evidence from three studies points to children/adolescents benefiting directly and indirectly from social support networks; directly, through their own networks, (Newman 2007; Wen 2008) and indirectly, through their parents’ networks (Harpham et al. 2006). Attendance at religious services (Parcel and Dufur 2001; Wen 2008), attending a school with a higher quality environment (Parcel and Dufur 2001) and living in a neighbourhood with higher levels of safety (Dorsey and Forehand 2003) were all associated with fewer general internalising/externalising problems.

In sum, positive relationships that exist within the family and those that extend out into the community are associated with better outcomes when internalising and externalising behaviours are assessed as a composite. Children and adolescents also seem to benefit from the structural support that comes in the form of higher quality schools and neighbourhoods.

### Mental health and behavioural problems – synthesis

The synthesised results showing the role and impact of family and community social capital across the full set of outcomes are presented in Table 1. There were a total of 172 investigated associations in the 55 included studies: 84 of these associations were positive, showing higher levels of social capital to be associated with better child/adolescent outcomes; 7 were negative, showing higher levels of social capital to be associated with poorer outcomes; and, in 51 cases no association was identified between social capital and the outcome.

**Table 1:** **Evidence table showing pattern of investigated associations between social capital and mental health/behavioural problems**

Association	Family structure	Parent–child relations	Adult interest	Parental monitoring	Extended family support	Composite/Other family social capital	Social support networks	Civic engagement	Trust & safety	Religiosity	Quality of school	Quality of neighbourhood	Composite/Other community social capital	Total
Number of investigated associations	10	25	3	9	6	10	33	9	5	9	13	28	12	172
Positive	3	16	2	3	2	9	17	2	2	4	6	13	5	84
Negative	1			4			1					1		6
None	5	2	1	2	3	1	7	7	2	2	3	10	6	51
Sub-group differences		4			1		4			3	4	3		19
Inconclusive results	1	3					4		1			1	1	11

## Discussion

### Summary of results

The primary aim of this integrative systematic review was to identify, analyse and synthesise empirical evidence on the association between family and community social capital and mental health and behavioural problems in children and adolescents. In doing so we assessed evidence from 55 studies making this the largest and most comprehensive systematic review in this field. In addition, this is, to the best of our knowledge, the first review to focus specifically on the mental health/behavioural problems of children and adolescents.

The large body of evidence included in this review supports the conclusion that both FSC and CSC are important in the context of children and adolescents’ mental health and behavioural problems.

#### Family social capital

In the case of FSC, parent–child relationships offered the most consistent protective role for children and adolescents, with the majority of the observed associations being in the positive direction. Parent–child relationships characterised by, for example, positive communication (Birndorf et al. 2005), feelings of nurturance (Yugo and Davidson 2007), support (Springer et al. 2006), and low levels of conflict (Ying and Han 2008) were associated with fewer reported mental health and behavioural problems in the children/adolescents. There was no evidence to suggest that positive parent–child relationships are detrimental to children or adolescents’ mental health and/or behaviours; however, some sub-groups of children/adolescents seem to derive more benefit than others from positive relationships with their parents (Glendinning and West 2007; Kliewer et al. 2004). The protective role of the parent–child relationship is well documented in relation to other outcomes. For example, parent–child relations characterised by appropriate control and high levels of responsiveness to the child’s needs (i.e. authoritative parenting) have been shown to be protective against adolescent health risk behaviours (Newman et al. 2008; Piko and Balázs 2012) and promote better educational outcomes (Dornbusch et al. 1987). Thus, is it important that evidence-based early interventions designed to foster positive parent–child relationships, such as the Triple P – Positive Parenting Program (Sanders 2008) are made available and accessible to families.

FSC that extends beyond the parent–child relationship to wider family relationships also appears to protect children/adolescents from developing mental health/behavioural problems, or it supports them in achieving better outcomes. Children and adolescents from families that are cohesive (Ying and Han 2008), high in justice (i.e. fairness) and trust (Delsing et al. 2005) and where members spend more time together (Fulkerson et al. 2006) had better mental health and behavioural outcomes. The role of the extended family has previously been highlighted as an important social capital resource in the adult literature; bonding forms of capital are generated and exploited in the intra-family relationships and families can bridge individual members to wider social resources. For example, in comparison to healthy adults, adults with psychiatric disorders perceive themselves as having less meaningful relationships with family members, their family connections are fewer in number (i.e. has fewer extended family members) and they report that their families are less cohesive (Widmer et al. 2008). However, it is unclear whether individuals with limited access to FSC are at higher risk of developing mental health and/or behavioural problems or whether families where problems exist have limited capacity to generate and exploit social capital because of the consequences of the mental ill-health/behavioural problems. Research designed to understand the direction of association/causation, particularly in children and adolescents, is essential if interventions that capitalise on the beneficial effects of social capital are to be developed to support better outcomes.

The evidence for parental monitoring was inconsistent, with almost equal numbers of the associations being reported as positive (Parcel and Dufur 2001; Ying and Han 2008) and negative (Glendinning and West 2007; Maynard and Harding 2010; Yugo and Davidson 2007). This means that in some circumstances increased parental monitoring may be associated with poorer mental health/behavioural problem outcomes. However, in this review, we were unable to identify any trends within the data that might explain when parental monitoring works to support better mental health/behavioural problem outcomes and when it presents as a risk factor. Research in other areas of the literature suggests that poorer mental health/behavioural problem outcomes may be a consequence of parenting styles associated with harsh discipline (e.g. authoritarian parenting) and which might be interpreted by the young person as over-controlling (Thompson et al. 2003). In the context of social capital, this parental over-control may threaten the young person’s sense of autonomy and control over their own lives and limit their ability to generate and mobilise their own social capital (James and Prout 1997).

Half of the studies that examined family structure failed to find a significant relationship with mental health and problem behaviour outcomes. However, the studies that did reported significant results suggested that youth who live in two-parent households have more positive outcomes. These findings echo those published elsewhere in the literature; for example, a number of studies have highlighted the protective role that family structure can have for young people in terms of sexual health (Kerrigan et al. 2006; Wight et al. 2006) and substance misuse (Winstanley et al. 2008).

#### Community social capital

In the context of CSC, the weight of evidence points to children and adolescents benefiting from social support networks. Children and adolescents report fewer mental health and behavioural problems when they have wider social support networks of peers (Rotenberg et al. 2004) and non-familial adults (DuBois et al. 2002) and when their social support networks are higher in quality (Bosacki et al. 2007; Ciairano et al. 2007; Windle 1994). This reinforces a large body of literature that illustrates the importance of social networks across a wide range of life domains, including educational attainment (Eggens et al. 2008). Providing safe and enriching opportunities for children and young people to extend and exploit their own social support networks should, therefore, be an important goal for policy makers and practitioners.

Young people, especially younger children, also appear to accrue indirect benefit from their parents having wider and higher quality social support networks (Beiser et al. 2011; El-Dardiry et al. 2012). Demonstrating that children and adolescents can achieve health benefits through their own social resources and through social resources accumulated by significant others (e.g. parents) has important implications when considering the targeting and reach of social capital interventions. For example, interventions that focus on enhancing support networks for parents of children with chronic health conditions have been shown to be effective in eliciting positive child and parent outcomes (Chernoff et al. 2002; Ireys et al. 2001). The evidence in this review suggests that, rather than focusing in sub-populations of parents, increasing access to interventions that help parents develop their support networks may be beneficial for all families.

There was little evidence to suggest that civic engagement was associated with mental health and behaviour problems. On the other hand, attending a school with a higher quality environment (e.g. feeling school is a safe place to be) (Bearinger et al. 2005; Beiser et al. 2011; Birndorf et al. 2005; Young et al. 2011; Yugo and Davidson 2007) and living in a high quality neighbourhood (e.g. having fewer hazards and higher levels of informal social control) (Aneshensel and Sucoff 1996, Beiser et al. 2011; Drukker et al. 2003) were both associated with better mental health and fewer problem behaviours.

More frequent attendance at religious services was also found to be related to better mental health/fewer behavioural problem outcomes (Abbotts et al. 2004; Oman et al. 2005; Parcel and Dufur 2001). It is important to note, however, that there was no evidence that personal importance of religion or religiosity was associated with the outcomes. The measures employed in the relevant studies assessed frequency of attendance or participation in religious services and we hypothesise that religious participation is a proxy‒indictor of social support networks and that participation in these groups may facilitate the development of bonded social support networks that can offer culturally appropriate support to young people and their families (Smith 2003). Further more nuanced research is needed to help illuminate the association between religion/religious service attendance and mental health/behavioural problem outcomes in children and adolescents.

Overall, we found little evidence of the elements of CSC being related to poorer outcomes; however, we did find evidence that both FSC and CSC can have a differential effect on some sub-groups of children and adolescents and a number of relationships require further exploration. For example, positive parent–child relationships appear to be important for supporting better mental health/behavioural problem outcomes for children and adolescents living in low violence neighbourhoods but this is not replicated for young people living in neighbourhoods high in violence (Kliewer et al. 2004). Schools may provide a source of capital that is important in supporting self-esteem/worth in adolescents from urban communities but not those living in rural communities (Glendinning and West 2007). In addition, neighbour-based parental support networks are associated with fewer behavioural problem outcomes in preschool children living in affluent neighbourhoods but increased behavioural problems in impoverished neighbourhoods (Caughy et al. 2003). It is, therefore, important that future research seeks to uncover the mechanisms through which social capital may exert different influences on the mental health (including behavioural problems) of children and adolescents living in different contexts.

### Strengths and limitations

The strengths and limitations of this review exist at two levels: in the review process itself and the individual studies. In terms of the review process, while the extensive heterogeneity in the outcome measures prevented us from performing a meta-analysis, by adopting an integrative approach with a robust analytical process we were able to synthesis data from a large number of studies that employed a range of different research designs. Moreover, with the exception of one, the included studies were rated by the reviewers as being moderate to high quality which strengthens the conclusions that can be drawn from the synthesised results. We do, however, acknowledge that relevant literature may not have been identified. For example, journals are known to favour papers that report statistically significant results meaning that studies failing to identify significance may be under-represented. Our success in capturing studies was also dependent upon adequate indexing of papers within the databases; however, as outlined above, we did take measures to ensure that our search strategy was as robust as possible.

In terms of the individual studies, social capital is a multifaceted concept whose dimensions function in various directions (Morrow 1999; Stone 2001), the lack of an agreed definition and little uniformity in its measurement across the studies made synthesising the evidence challenging. However, we employed well-defined study-specific inclusion/exclusion criteria to ensure relevant data were captured in an objective fashion. It is important that future research defines and operationalises social capital in a more consistent and robust manner enabling a clearer understanding of its relationship to important outcomes and assisting comparisons across studies.

Other reviews exploring social capital and mental health have included children and adolescents but considered them alongside adults (Almedom 2005; De Silva et al. 2005) and, as we note above, this means that the definitions of social capital applied in these previous syntheses of data may not be adequate for the child/adolescent group (Morgan 2011; Morrow 2001). We sought to overcome this criticism of previous work by adopting a definition of social capital that was theoretically pluralistic but informed by previous research to ensure that it encapsulated aspects of social capital relevant to young people. For example, given that school is integral to the lives of children and adolescents and their families, and in keeping with the work of Coleman (1988) and Ferguson (2006), we included this in our definition of community social capital.

Moreover, as noted in the Background section, while current directions in health and social policy place emphasis on the promotion of an assets based approach, the majority of research in the field of mental health/behavioural problems in children and adolescents is conducted at the negative end of the spectrum, focusing on the onset and treatment of disorder. That said, while there was limited availability of positively framed evidence, we sought to ensure that, where possible, the conclusions we drew were framed within an assets based approach.

Despite the limitations that we highlight, the large body of evidence in this review means that we have been able to demonstrate conclusively that FSC and CSC are both associated with mental health and behavioural problems in children and adolescents; however the cross sectional nature of many of the studies prevented us from drawing firm conclusions about the direction of these associations. While it is assumed that social capital can be an asset that supports better outcomes, it is equally plausible that families with a child/adolescent who has a mental health problem (including behavioural problems) are limited in their ability to access and mobilise social capital. This review also highlights a lack of qualitative evidence. Future qualitative research is essential if we are to develop a theoretical framework that articulates the mechanisms through which social capital works to effect health and wellbeing, the various circumstances in which this occurs and how family and community social capital interact and mediate outcomes.

## Conclusions

To the best of our knowledge, this is the first review to focus exclusively on the relationships that exist between family and community social capital and mental health and behavioural problems in children and adolescents. Our comprehensive examination of the available evidence suggests that there are important ways in which social capital, generated and mobilised at the family and community level, can influence the mental health and problem behaviours of young people. Thus this review contributes to the early years’ mental health/behavioural problem literature and also the social capital evidence base, which has been criticised for being too adult-centric (Morrow 2004).

The present findings highlight the need for researchers to develop a robust theoretical framework that fully articulates the relationships that exist between social capital and mental health and behavioural problems, especially in the context of children and adolescents. Furthermore, we have made explicit the potential for young people to generate and exploit their own social capital to promote better health outcomes. Understanding the mechanisms through which this can occur would support policy makers to embed social capital as an underpinning feature of public health policies and interventions (Gillies 2009; World Health Organization 2002).

Current international strategies, such as Health 2020 (World Health Organization 2012), call for early interventions to build resilience in communities and develop people-centred health systems to reduce health inequality and promote better mental health outcomes (including behavioural problems) for future generations (World Health Organization 2003; World Health Organization 2012; World Health Organization Regional Office for Europe 2012). However, it must be acknowledged that, the current economic crisis has necessitated that international governments implement measures to reduce budget deficits. These austerity measures include cuts to public spending, services and benefits which will significantly impact on what services and resources are available/feasible to support families (Joseph Rowntree Foundation 2013). Interventions that build on the strengths/assets of families and communities, including their social capital, and which encourage families, communities and outside agencies to work together to ‘co-produce’ solutions would appear timely.

## Electronic supplementary material

Additional file 1:Search strategy (PsycINFO).(PDF 378 KB)

Additional file 2: Table S1: Description of studies included in the review (ordered by outcome). (PDF 427 KB)

Below are the links to the authors’ original submitted files for images.

Authors’ original file for figure 1
