# Supplementary material for: The association between social capital and mental health and behavioural problems in children and adolescents: an integrative systematic review
Source: BMC Psychol. 2014 Mar 26;2:7. doi: 10.1186/2050-7283-2-7 (PMC4270040; doi:10.1186/2050-7283-2-7)
Supplement: Supplementary file 1 — Additional file 1:Search strategy (PsycINFO).(PDF 378 KB) [file 40359_2013_9034_MOESM1_ESM.pdf]

Additional File 1: Search strategy (PsycINFO[1])

Topic	Index terms and keywords
**Social capital**	S1 social networks/ OR online social networks/<br>S2 social support/<br>S3 friendship/<br>S4 friend*<br>S5 peer relations/ OR peer pressure/<br>S6 family relations/ OR child discipline/ OR childrearing practices/ OR family conflict/ OR marital relations/ OR parent child relations/ OR parental role/ OR sibling relations<br>S7 intergenerational relations/<br>S8 parenting/ OR authoritarian parenting/ OR childrearing practices/ OR parent child communication/ OR parental involvement/ OR parenting style/ OR permissive parenting/<br>S9 school environment/<br>S10 school*<br>S11 community involvement/<br>S12 neighborhoods/ OR ghettoes/<br>S13 social structure/<br>S14 social capital/<br>S15 social capital<br>S16 salutogenesis OR asset* based OR health asset*<br>S17 trust (social behaviour)/ OR reciprocity/<br>S18 trust OR reciprocity<br>S19 social cohesion OR neighbourhood cohesion<br>S20 family social capital OR family capital<br>S21 community social capital OR community capital<br>S22 community processes/<br>S23 OR/1-22
**Psycho-social health and wellbeing**	S24 mental health/ OR community mental health/<br>S25 emotional intelligence/<br>S26 psychological development/<br>S27 cognitive development/ OR intellectual development/ OR language development/ OR language delay<br>S28 emotional development/<br>S29 psychosocial development/ OR childhood play/ OR psychosexual development/<br>S30 health behavior/<br>S31 well being/<br>S32 quality of life/<br>S33 emotional adjustment/ OR emotional control/ OR identity crisis/<br>S34 public health/<br>S35 health promotion/<br>S36 behavior problems/<br>S37 child psychology/ OR adolescent psychology/<br>S38 adolescent development/<br>S39 childhood development/ OR early childhood development/ OR proximal development

[1]). The search strategy was appropriately tailored for the other eight databases searched.

	S40	OR/24-39
**Combined search**	S41	S23 AND S40
**Combined search with delimiters**	S42	limit S41 as follows:<br>publication year – 1990-2012<br>peer reviewed journal articles<br>English language<br>age groups – neonatal (birth-1 month), infancy (2-23 months), preschool (2-5 years), school age (6-12 years), adolescence (13-17 years)<br>population group – humans
