# Supplementary material for: The association between social capital and mental health and behavioural problems in children and adolescents: an integrative systematic review
Source: BMC Psychol. 2014 Mar 26;2:7. doi: 10.1186/2050-7283-2-7 (PMC4270040; doi:10.1186/2050-7283-2-7)
Supplement: Supplementary file 2 — Additional file 2: Table S1: Description of studies included in the review (ordered by outcome). (PDF 427 KB) [file 40359_2013_9034_MOESM2_ESM.pdf]

Additional file 2: Table S1

Table S1: Description of studies included in the review (ordered by outcome)

Authors, Country<br>Quality rating	Design	Sample	FSC	CSC	Outcomes
**1) Self-esteem and self-worth**					
Abbotts et al 2004<br>UK<br>high quality	cross-sectional survey (West of Scotland 11 to 16 Study)	size: 1,690<br>age group: adolescents<br>age range: 11 yrs<br>sex: not stated<br>ethnic group: not stated	✗	✓	Weekly church attendance was associated with higher self-esteem in Catholic adolescents but lower self-esteem in Church of Scotland (Protestant) adolescents.
Aim: To examine the relationship between church attendance and self-esteem.					
Birndorf et al 2005<br>USA<br>moderate quality	prospective longitudinal cohort study (National Education Longitudinal Survey)<br>data collected 1988-1992	size: 16,489<br>age group: adolescents<br>age range: 13-18 yrs<br>sex: 50% female<br>ethnic group: 72% White	✓	✓	Positive parent-adolescent communication and feeling safe at school at baseline predictive of higher self-esteem 3yrs later. Religious participation was predictive for males only. No role for parental monitoring, adult interest in the child or social support networks.
Aim: To examine those modifiable community and individual factors that may influence high self-esteem over time.					

Ciarano et al 2007<br>Italy<br>high quality	longitudinal survey	size: 622<br>age group: adolescents<br>age range: 14-20 yrs<br>sex: 52% female<br>ethnic group: not stated	×	✓	Adolescents with friendships high in support reported positive self-perception. When friendship quality improved over time so did positive self-perception.
Aim: To distinguish different patterns of friendships, in terms of support and conflict, and reciprocity of friendship (and any link to positive self-perception).					
Drukker et al 2006<br>Netherlands<br>high quality	longitudinal cohort study<br>data collected 2000-03, Time 1 (T1), Time 2 (T2)	size: 475<br>age group: adolescents<br>mean age T1: 11 yrs<br>mean age T2: 13.5 yrs<br>sex: 52% female<br>ethnic group: not stated	×	✓	No role for community social capital.
Aim: To study associations between neighbourhood environment and changes in health related quality of life (including self-esteem).					
DuBois et al 2002<br>USA<br>moderate quality	controlled trial<br>1 intervention group<br>1 control group	size: 134<br>age group: children & adolescents<br>age range: 7-15 yrs<br>sex: 55% female<br>ethnic group: 63% Black	×	✓	Self-esteem in the intervention group greater than in the control group post-intervention.
Aim: To evaluate the effects of a mentoring programme on self-esteem.					

El-Dardiry et al 2012<br>Greece<br>high quality	cross-sectional survey	size: 542<br>age group: children & adolescents<br>age range: 8-12 yrs<br>sex: 53% female<br>ethnic group: not stated	×	✓	Higher levels of parental social support and perceptions of neighbourhood social capital associated with more positive self-perception.
Aim: To examine the potential association between social capital and child health-related quality of life (including self-perception).					
Glendinning et al 2007<br>Serbia<br>moderate quality	cross-sectional survey	size: 647<br>age group: adolescents<br>age range: 14-15 yrs<br>sex: 53%<br>ethnic group: not stated	✓	✓	Feeling supported by parents was protective for rural adolescents. Higher quality school was protective in urban adolescents. Higher peer support associated with higher self-esteem. Higher parental monitoring was a risk for all and community constraint a risk for rural adolescents only.
Aim: To examine young people's life circumstances and the implications for mental health (including self-esteem).					
Jager et al 2011<br>USA<br>moderate quality	longitudinal survey (ADD Health)<br>data collected 1994-2002	size: 4,233<br>age group: adolescents<br>age range: 13-19 yrs<br>sex: not stated<br>ethnic group: not stated	✓	✓	Different patterns of positive relationships with others share different relationships with self-esteem.
Aim: To examine heterogeneity in adolescent relationship constellations and its relation to adolescent adjustment (including self-image).					

Ying et al 2008<br>USA<br>high quality	cross-sectional survey (Children of Immigrants Longitudinal Study)	size: 491<br>age group: adolescents<br>mean age: 17.4 yrs<br>sex: 50% female<br>ethnic group:100% South East Asian American	✓	×	Parent-child relationships high in cohesion and low in conflict were associated with better self-esteem.
Aim: To examine the contribution of parental acculturation, parental involvement, and intergenerational relationships to wellbeing (including self-esteem).					
Yugo et al 2007<br>Canada<br>low quality	cross-sectional survey (National Longitudinal Survey of Children & Youth)	size: 3,725<br>age group: adolescents<br>age range: 12-15 yrs<br>sex: not stated<br>ethnic group: not stated	✓	✓	Higher parental nurturance, peer connectedness and school engagement associated with increased odds of high self-worth. Higher parental monitoring associated with reduced odds of high self-worth. No role for civic engagement.
Aim: To examine which of five assets accounts for the most variance in positive health outcomes (including self-worth).					

**2) Internalising behaviours**					
Abbotts et al 2004<br>UK<br>high quality	cross-sectional survey (West of Scotland 11 to 16 Study)<br>data collected 1994	size: 1,690<br>age group: adolescents<br>age range: 11 yrs<br>sex: not stated<br>ethnic group: not stated	×	✓	Weekly church attendance associated with lower levels of depression, malaise & social anxiety in Catholic adolescents, and higher levels in Church of Scotland (Protestant) adolescents.
Aim: To examine the relationship between church attendance and social anxiety and depression in children from Christian denominations.					
Aneshensel et al 1996<br>USA<br>high quality	cross-sectional survey	size: 877<br>age group: adolescents<br>age range: 12-17 yrs<br>sex: 47% female<br>ethnic group: 49% Hispanic	✓	✓	Higher levels of neighbourhood ambient hazards associated with increased depression and anxiety. Higher social cohesion associated with lower depression only.
Aim: To examine how structural aspects of neighbourhood context affect adolescent emotional wellbeing (including depressive symptoms and anxiety).					

Beiser et al 2011<br>Canada<br>moderate quality	cross-sectional survey (New Canadian Children & Youth Survey)<br>data collected 2002-04	size: 2,031<br>age group: children and adolescents<br>age range: 4-6 yrs and 11-13 yrs<br>sex: 49% female<br>ethnic group: 100% mainland Chinese, Hong Kong Chinese and Filipino immigrants	×	✓	Increased parents' social support, perceived quality of school and neighbourhood, and parents speaking native language of migrant country associated with fewer emotional problems.
Aim: To explore the extent social capital accounts for the relationship between immigrant children's mental health (including emotional problems) and place of resettlement.					
Bosacki et al 2007<br>Canada<br>high quality	cross-sectional survey	size: 7,290<br>age group: adolescents<br>age range: 13-18 yrs<br>sex: 51% female<br>ethnic group: not stated	×	✓	Better quality friendships and lower social isolation associated with lower levels of depression and social anxiety.
Aim: To examine the connection between adolescent peer relationships and social anxiety and depression.					

Bowker et al 2010<br>USA<br>high quality	quasi-experiment	size: 156<br>age group: adolescents<br>age range: 10-15 yrs<br>sex: 51% female<br>ethnic group: 77% White	×	✓	Higher conflict friendships associated with internal blame attributions in overweight, but not normal weight, adolescents. Positive friendship characteristics associated with more adaptive coping patterns in overweight, but not normal weight, adolescents.
Aim: To explore the influence of friendship on psychological wellbeing in overweight and normal weight adolescents.					
Caughy et al 2003<br>USA<br>high quality	cross-sectional survey	size: 200<br>age group: preschool<br>age range: 3-4.5 yrs<br>sex: 54% female<br>ethnic group: 100% Black	×	✓	Higher quality of neighbourhood associated with fewer internalising problems. Support from neighbours protective against internalising problems in affluent neighbourhoods but a risk factor in impoverished neighbourhoods.
Aim: To examine the impact of parents' attachment to community as a predictor of the mental health status of their children.					
Caughy et al 2006<br>USA<br>high quality	cross-sectional survey	size: 241<br>age group: children<br>mean age: 6.6 yrs<br>sex: 49% females<br>ethnic group: 92% Black	×	✓	Negative neighbourhood climate, but not cohesion and control, associated with more internalising problems.
Aim: To examine whether racial socialization and child outcomes (including internalised behaviours) were consistent across varied residential neighbourhood contexts.					

Caughy et al 2008<br>USA<br>high quality	cross-sectional survey	size: 405<br>age group: children<br>age range: 6-7 yrs<br>sex: 55%<br>ethnic group: 55% Black	✓	✓	Positive parent-child relations and better neighbourhood climate were protective. Higher neighbourhood control was protective in impoverished neighbourhoods only.
Aim: To examine whether neighbourhood social process characteristics are associated with differences in child behaviour problems (including internalising behaviour).					
Ciarano et al 2007<br>Italy<br>high quality	longitudinal survey	size: 622<br>age group: adolescents<br>age range: 14-20 yrs<br>sex: 52% female<br>ethnic group: not stated	✗	✓	Supportive friendships at baseline predictive lower levels of depression 6 months later.
Aim: To distinguish different patterns of friendships, in terms of support and conflict, and reciprocity of friendship and how this was associated with depression.					
Delsing et al 2005<br>Netherlands<br>high quality	cross-sectional survey	size: 576<br>age group: adolescents<br>age range: 11-16 yrs<br>sex: 51% female<br>ethnic group: 100% Dutch	✓	✗	Lower levels of justice and trust within family relationships associated with more internalising problems.
Aim: To examine the relationship between perceived justice and trust within family relationships and internalising problems.					

Drukker et al 2003<br>Netherlands<br>high quality	longitudinal cohort study	size: 563<br>age group: adolescents<br>age range: 10-12 yrs<br>sex: 51% female<br>ethnic group: not stated	×	✓	Neighbourhood informal social control, but not cohesion and trust, was protective in the context of mental health.
Aim: To study the associations between social capital and quality of life and behaviour (including mental health).					
Drukker et al 2006<br>Netherlands<br>high quality	longitudinal cohort study<br>data collected 2000-2003 - Time 1 (T1) and Time 2 (T2)	size: 475<br>age group: adolescents<br>mean age T1: 11 yrs<br>mean age T2: 13.5 yrs<br>sex: 52% female<br>ethnic group: not stated	×	✓	Neighbourhood factors did not predict changes in mental health over time.
Aim: To study associations between neighbourhood environment and changes in health related quality of life (including mental health).					
DuBois et al 2002<br>USA<br>moderate quality	controlled trial<br>1 control and 1 intervention group<br>mentoring intervention	size: 134<br>age group: children and adolescents<br>age range: 7-15 yrs<br>sex: 55% female<br>ethnic group: 63% Black	×	✓	No significant difference in emotional problems in the intervention group, compared to the control group, post-intervention.
Aim: To evaluate the effects (including on emotional problems) of a mentoring programme.					

El-Dardiry et al 2012<br>Greee<br>high quality	cross-sectional survey	size: 542<br>age group: children & adolescents<br>age range: 8-12 yrs<br>sex: 53% female<br>ethnic group: not stated	✗	✓	Higher levels of parental social support and neighbourhood social capital associated with more positive moods and emotions.
Aim: To examine the potential association between social capital and child health-related quality of life (including moods and emotions).					
Fitzpatrick et al 2005<br>USA<br>high quality	cross-sectional survey	size: 1,538<br>age group: adolescents<br>age range: 10-18 yrs<br>sex: 51% female<br>ethnic group: 100% Black	✓	✓	Higher composite family and community social capital were associated with lower depression scores.
Aim: To examine depressive symptomatology focusing on the role of capital as both personal and social resources.					
Fulkerson et al 2006<br>USA<br>high quality	cross-sectional survey<br>data collected 1996-97	size: 98,340<br>age group: adolescents<br>age range: 11-17 yrs<br>sex: 50% female<br>ethnic group: 86% White	✓	✗	Regular family meals (an indicator of family cohesion) associated with reduced odds of experiencing depressive/suicidal thoughts.
Aim: To examine the associations between family meal frequency, developmental assets and high-risk behaviours (including depression & suicide risk/attempts).					

Glendinning et al 2007<br>Serbia<br>moderate quality	cross-sectional survey	size: 647<br>age group: adolescents<br>age range: 14-15 yrs<br>sex: 53% female<br>ethnic group: not stated	✓	✓	Family and peer support protective for rural, but not urban, adolescents. School environment was protective only for urban adolescents. Parental and community monitoring were risk factors for both groups. No role for family structure.
Aim: To examine young people's life circumstances and the implications for mental health (including depressive mood).					
Jager 2011<br>USA<br>moderate quality	longitudinal survey (ADD Health)<br>data collected 1994-2002	size: 4,233<br>age group: adolescents<br>age range: 13-19 yrs<br>sex: not stated<br>ethnic group: not stated	✓	✓	Different patterns of positive relationships with others share different relationships with depressive effect.
Aim: To examine heterogeneity in adolescent relationship constellations and its relation to adolescent adjustment (including depressive affect).					
Kliewer et al 2004<br>USA<br>high quality	longitudinal survey	size: 69<br>age group: children and adolescents<br>age range: 9-13 yrs<br>sex: 45% female<br>ethnic group: 98% Black	✓	✓	Perceived positive relationship with caregiver protective for children/adolescents from low violence, but not high violence, communities. Observed parent-child relations not significant. Higher neighbourhood cohesion associated with fewer internalising problems.
Aim: To examine different levels of children's ecology that could protect youth from adjustment difficulties (including internalising symptoms).					

Landstedt et al 2009<br>Sweden<br>moderate quality	qualitative study<br>focus groups	size: 29<br>age group: adolescents<br>age range: 16-19 yrs<br>sex: not stated<br>ethnic group: not stated	✓	✓	Narrative described relationships with friends, family, teachers and classmates as important for better mental health.
Aim: To explore what students perceive as significant for mental health.					
Meltzer et al 2007<br>UK<br>moderate quality	cross-sectional survey	size: 3,340<br>age group: adolescents<br>age range: 11-16 yrs<br>sex: not stated<br>ethnic group: not stated	×	✓	Lower levels of neighbourhood trust and safety associated with higher reporting of emotional disorders.
Aim: To explore relationship between children's perceptions of trustworthiness and safety of their neighbourhoods and their mental health (including emotional disorders).					
Rasic et al 2011<br>Canada<br>moderate quality	cross-sectional survey	size: 1,615<br>age group: adolescents<br>age range: 15-19 yrs<br>sex: 49% female<br>ethnic group: not stated	×	✓	No role identified for religiosity.
Aim: To examine impact of social capital on the relationships between religion and depression and suicidal behaviour.					

Rotenberg et al 2004<br>UK and Canada<br>high quality	cross sectional & longitudinal survey<br>Study 1 (S1) UK<br>Study 2 (S2) Canada	size: 434<br>age group (S1): children<br>age group S2):adolescents<br>age range (S1): 9-11<br>age range (S2): 11-14 yrs<br>sex (S1): 53% female<br>sex (S2): 48% female<br>ethnic group (S1/S2): not stated	×	✓	Lower number of friendships associated with loneliness in cross-sectional analysis but did not predict longer term psychological adjustment.
Aim: To examine whether trustworthiness contributes positively to children's and adolescents' social relationship and psychological adjustment (including depressive symptoms and loneliness).					
Rotenberg et al 2005<br>UK<br>moderate quality	cross-sectional & longitudinal survey	size: 434<br>age group: children and adolescents<br>age range: 9-11 yrs<br>sex: not stated<br>ethnic group: not stated	×	✓	Very high and very low levels of trust in peers were associated with poorer mental health outcomes.
Aim: To assess the relationship between children's trust in peers, social exclusion, self-perceived social acceptance and internalising maladjustment (including depression, anxiety and loneliness) over 8 months.					

Springer et al 2006<br>El Salvador<br>high quality	cross-sectional survey	size: 930<br>age group: adolescents<br>age range: 12-19yrs<br>sex: 48% female<br>ethnic group: not stated	✓	✓	Low parental support associated with increased odds of suicidal thoughts. Low levels of social cohesion in school associated with increased risk of suicidal thoughts in females only.
Aim: To examine perceived parental social support and social cohesion at school with the prevalence of a range of youth risk behaviours (including depression, suicidal thoughts and attempts).					
Stevenson 1998<br>USA<br>high quality	cross-sectional survey	size: 160<br>age group: adolescents<br>mean age: 14.6 yrs<br>sex: 54% female<br>ethnic group: 100% Black	✓	✓	In high social capital neighbourhoods low kinship support associated with lower depression. In high risk, but not low risk neighbourhoods, neighbourhood social capital is protective.
Aim: To investigate impact of neighbourhood and kinship social support and fear of violence on emotional development (including depression).					
Wang et al 2011<br>Taiwan<br>moderate quality	cross-sectional survey	size: 453<br>age group: adolescents<br>age range: 12-16 yrs<br>sex: 46% female<br>ethnic group: not stated	✓	✓	No role for family or community social capital in promoting better stress management.
Aim: To examine the relationship between developmental assets and health promoting behaviour of adolescents (including ability to manage stress).					

Windle 1994<br>USA<br>high quality	longitudinal cohort study	size: 1,098<br>age group: adolescents<br>mean age: 16.2 yrs<br>sex: 52% female<br>ethnic group: 98% White	×	✓	Friendships high in hostility and low in reciprocity associated with increased depressive symptoms and suicidal behaviours in cross-sectional analyses but this did not impact on long-term outcomes.
Aim: To study concurrent and prospective relations between friendship characteristics and adolescent internalising problems (including depressive symptoms, suicidal thoughts and attempts).					
Xue et al 2005<br>USA<br>high quality	longitudinal cohort study (Project on Human Development in Chicago Neighborhoods)<br>data collected 1995-98	size: 2,805<br>age group: children<br>age range: 5-11 yrs<br>sex: 50% female<br>ethnic group: 48% Hispanic	×	✓	Children in neighbourhoods with higher levels of civic participation had better internalising scores. No role identified for collective efficacy.
Aim: To examine associations between neighbourhood structural characteristics and children's mental health (including internalising behaviours).					
Ying et al 2008<br>USA<br>high quality	cross-sectional survey (Children of Immigrants Longitudinal Survey)	size: 491<br>age group: adolescents<br>mean age: 17.4 yrs<br>sex: 50% female<br>ethnic group: 100% Southeast Asian American	✓	×	Adult interest in the child and parental monitoring predicted parent-child relationships and parent-child relationship high in cohesion and low in conflict associated with better child outcomes (including lower levels of depression).
Aim: To examine the contribution of parental acculturation, parental involvement, and intergenerational relationships to wellbeing (including depressive symptoms).					

Young et al 2011<br>UK<br>high quality	longitudinal survey (West of Scotland 11 to 16 survey)<br>data collected 1994-2004<br>Time 1 (T1), Time 2 (T2), Time 3 (T3)	size: 1,698<br>age group: adolescents<br>mean age T1: 11 yrs<br>mean age T2: 15 yrs<br>mean age T3: 19 yrs<br>sex: 50% female<br>ethnic group: not stated	×	✓	Mis-match between pupil's religion and the denomination of the school they attended increased risk of future suicide attempts and self-harm behaviours. Poorer-rated schools were associated with suicide risk in future. No role for quality of neighbourhood.
Aim: To explore the association between the school and suicide-risk, adjusting for important factors such as perception of local neighbourhood.					
**3) Externalising behaviours**					
Abbotts et al (2004)<br>UK<br>high quality	cross-sectional survey<br>data collected 1994	size: 1,690<br>age group: adolescents<br>age range: 11 yrs<br>sex: not stated<br>ethnic group: not stated	×	✓	Weekly church attendance was associated with lower aggression scores in Catholic and Church of Scotland (Protestant) adolescents.
Aim: To examine the relationship between church attendance and aggressive behaviour in children from Christian denominations.					

Aneshensel et al 1996<br>USA<br>high quality	cross-sectional survey	size: 877<br>age group: adolescents<br>age range: 12-17 yrs<br>sex: 47% female<br>ethnic group: 49% Hispanic	✓	✓	Neighbourhoods high in hazards associated with poorer outcomes. Those living in a 1-parent household had higher oppositional defiant, but not conduct disorder symptoms. No role for social cohesion.
Aim: To examine how structural aspects of neighbourhood context affect adolescent emotional wellbeing (including conduct disorder and oppositional defiant disorder).					
Bearinger et al 2005<br>USA<br>moderate quality	cross-sectional survey (Urban Indian Youth Health Survey)<br>data collected 1995-98	size: 577<br>age group: children & adolescents<br>mean age:9-15 yrs<br>sex: 52% female<br>ethnic group: 100% American Indian	✓	✓	Adolescents who feel more connected to school had decreased odds of reporting violence perpetration. No role for extended family support or social support networks.
Aim: To identify the most salient risk and protective factors for violence perpetration.					
Caughy et al 2003<br>USA<br>high quality	cross-sectional survey	size: 200<br>age group: preschool<br>age range: 3-4.5 yrs<br>sex: 54% female<br>ethnic group: 100% Black	✗	✓	Support from neighbours was a risk factor in impoverished neighbourhoods only. No role for quality of neighbourhood.
Aim: To examine the impact of parents' attachment to community as a predictor of the mental health status of their children (including externalising behaviours).					

Caughy et al 2006<br>USA<br>high quality	cross-sectional survey	size: 241<br>age group: children<br>mean age: 6.6 yrs<br>sex: 49% females<br>ethnic group: 92% Black	✗	✓	No role for community social capital.
Aim: To examine whether racial socialization and child outcomes (including externalising behaviours) were consistent across varied residential neighbourhood contexts.					
Caughy et al 2008<br>USA<br>high quality	cross-sectional survey	size: 405<br>age group: children<br>age range: 6-7 yrs<br>sex: 55%<br>ethnic group: 55% Black	✓	✓	Positive parent-child relationship characteristics were protective, and negative characteristics a risk factor, in relation to externalising problems. No role for community social capital.
Aim: To examine whether neighbourhood social process characteristics are associated with differences in child behaviour problems (including externalising behaviour).					
Champion et al 2008<br>USA<br>moderate quality	cross-sectional survey	size: 2,090<br>age group: adolescents<br>age range: 14-19 yrs<br>sex: 49% female<br>ethnic group: 61% White	✓	✓	Positive parent-adolescent relationships, participation in extra-curricular activities and higher quality neighbourhood environment, but not neighbourhood connectedness, associated with decreased odds of reporting date fighting perpetration. No role for quality of school or trust and safety.
Aim: To explore contextual factors associated with date fight perpetration.					

Ciarano et al 2007<br>Italy<br>high quality	longitudinal survey	size: 622<br>age group: adolescents<br>age range: 14-20 yrs<br>sex: 52% female<br>ethnic group: not stated	✗	✓	Friendships high in support associated with lower levels of physical aggression but increased lying and disobedience.
Aim: To distinguish different patterns of friendships, in terms of support, conflict, reciprocity and how this impacted on adolescent psychosocial adjustment.					
Delsing et al 2005<br>Netherlands<br>high quality	cross-sectional survey	size: 576<br>age group: adolescents<br>age range: 11-16 yrs<br>sex: 51% female<br>ethnic group: 100% Dutch	✓	✗	Lower levels of justice and trust within families associated with more externalising problems.
Aim: To examine the relationship between perceived justice and trust within family relationships and externalising problems.					
Drukker et al 2003<br>Netherlands<br>high quality	cross-sectional cohort study	size: 563<br>age group: adolescents<br>age range: 10-12 yrs<br>sex: 51% female<br>ethnic group: not stated	✗	✓	Neighbourhood informal social control, but not cohesion and trust, was protective in the context of externalising problems.
Aim: To study the associations between social capital and quality of life and behaviour (including externalising behaviour).					

Drukker et al 2010<br>Netherlands<br>high quality	longitudinal cohort study<br>data collected 2002-03<br>Time 1 (T1), Time 2 (T2)	size: 672<br>age group: adolescents<br>age range (T1): 11 yrs<br>age range (T2): 13-14 yrs<br>sex: 52% female<br>ethnic group: not stated	×	✓	Females from neighbourhoods low in social cohesion and trust reported more delinquent behaviours but there was no role for informal social control. Males from neighbourhoods high in informal social control reported more delinquent behaviours but there was no role for cohesion and trust.
Aim: To investigate the influence of neighbourhood variables on delinquency.					
DuBois et al 2002<br>USA<br>moderate quality	controlled trial<br>1 intervention group (I)<br>1 control group (C)<br>mentoring intervention	size: 134<br>age group: children and adolescents<br>age range: 7-15 yrs<br>sex: 55% female<br>ethnic group: 63% Black	×	✓	No significant difference in behavioural problems in the intervention group, compared to the control group, post-intervention.
Aim: To evaluate the effects (including externalising behaviours) of a mentoring programme.					
El Hajj et al 2011<br>Lebanon<br>moderate quality	cross-sectional survey (Urban Health Survey)	size: 674<br>age group: adolescents<br>age range: 13-19 yrs<br>sex: 0% females<br>ethnic group: not stated	✓	✓	Adolescents with wider and better quality social networks more likely to report fighting. Neighbourhood environment presented some risk and some protective factors. No role for family social capital or civic engagement, trust or reciprocity.
Aim: To assess the relationship of social capital to physical fights in three disadvantaged neighbourhoods.					

Fulkerson et al 2006<br>USA<br>high quality	cross-sectional survey<br>data collected 1996-97	size: 98,340<br>age group: adolescents<br>age range: 11-17 yrs<br>sex: 50% female<br>ethnic group: 86% White	✓	×	Regular family meals (an indicator of family cohesion) were associated with reduced odd of violent behaviours.
Aim: To examine the associations between family meal frequency, developmental assets and high-risk behaviours (including violent behaviour).					
Jager 2011<br>USA<br>moderate quality	longitudinal survey (ADD Health)<br>data collected 1994-2002	size: 4,233<br>age group: adolescents<br>age range: 13-19 yrs<br>sex: not stated<br>ethnic group: not stated	✓	✓	Different patterns of positive relationships with others share different relationships with delinquent behaviours.
Aim: To examine heterogeneity in adolescent relationship constellations and its relation to adolescent adjustment (including delinquency).					
Johnson et al 1999<br>USA<br>high quality	cross-sectional survey (Choices of Life for Adolescent Success Study – CLASS)	size: 200<br>age group: adolescents<br>age range: 15-20 yrs<br>sex: 60% female<br>ethnic group: 100% Black	×	✓	No role for community social capital.
Aim: To examine the viability of a social systems perspective in predicting violence in youth.					

Kingston et al 2009<br>USA<br>high quality	cross-sectional survey (Denver Youth Survey)	size: 741<br>age group: adolescents<br>age range: 12-17 yrs<br>sex: not stated<br>ethnic group: not stated	✓	✓	Neighbourhoods characterised as limiting opportunity associated with increased reporting of property, but not violent, offending. No role for family structure, social support networks, social cohesion or institutional effectiveness.
Aim: To test the relationships between neighbourhood social structure, social processes, delinquent opportunity structures and rates of adolescent delinquency.					
Kliewer et al 2004<br>USA<br>high quality	longitudinal survey	size: 69<br>age group: children and adolescents<br>age range: 9-13 yrs<br>sex: 45% female<br>ethnic group: 98% Black	✓	✓	Perceived positive relationship with caregiver associated with fewer externalising behaviours. No role for community social capital.
Aim: To examine different levels of children's ecology that could protect youth from adjustment difficulties (including internalising symptoms).					
Meltzer et al 2007<br>UK<br>moderate quality	cross-sectional survey	size: 3,340<br>age group: adolescents<br>age range: 11-16 yrs<br>sex: not stated<br>ethnic group: not stated	×	✓	Lower levels of neighbourhood trust, but not safety, associated with higher reporting of conduct disorders.
Aim: To explore the relationship between children's perceptions of trustworthiness and safety of their neighbourhoods and mental health (including conduct disorder).					

Oman et al 2005<br>USA<br>moderate quality	cross-sectional survey<br>data collected 1999-2000	size: 1,2777<br>age group: adolescents<br>age range: 13-19 yrs<br>sex: 52% female<br>ethnic group: 48% White	✓	✓	Positive family communication protective for adolescents from 1-parent households. Social networks were protective for some indicators of violence and delinquency. Religious participation and having a peer/adult role model were protective for delinquency. No role for civic engagement.
Aim: To investigate how the relationships between youth assets related to aggressiveness and delinquency.					
Smith et al 2009<br>USA<br>low quality	cross-sectional survey	size: 61<br>age group: adolescents<br>age range: 11-13 yrs<br>sex: 51 % female<br>ethnic group: 53% Black	✓	✓	No role identified for family or community social capital.
Aim: To identify assets that contribute to positive youth outcomes, specifically health behaviours, thriving indicators, and not engaging in delinquency.					
Springer et al 2006<br>El Salvador<br>high quality	cross-sectional survey	size: 930<br>age group: adolescents<br>age range: 12-19yrs<br>sex: 48% female<br>ethnic group: not stated	✓	✓	Low parental support associated with increased odds of reporting aggression. Low levels of social cohesion in school associated with increased odds of reporting aggression in males only.
Aim: To examine perceived parental social support and social cohesion at school with the prevalence of a range of youth risk behaviours (including aggression & violence).					

Stevenson 1997<br>USA<br>high quality	cross-sectional survey	size: 202<br>age group: adolescents<br>age range: 14-15 yrs<br>sex: 63% female<br>ethnic group: 100% Black	✓	✓	High kinship support was associated with anger suppression in high-, but not low- risk neighbourhoods.
Aim: To assess the relationship between sense of neighbourhood, social support, kinship or family social support and anger experience and expression.					
Windle 1994<br>USA<br>high quality	longitudinal cohort study	size: 1,028<br>age group: adolescents<br>mean age: 16.2 yrs<br>sex: 52% female<br>ethnic group: 98% White	×	✓	Friendships high in hostility associated with increased delinquency behaviours in cross-sectional analyses but did not predict longer-term delinquency.
Aim: To study concurrent and prospective relations between friendship characteristics and adolescent externalising behavioural problems (including delinquency).					
**4) Composite internalising and externalising behaviours**					
Dorsey et al 2003<br>USA<br>high quality	cross-sectional survey	size: 130<br>age group: children & adolescents<br>age range: 7-15 yrs<br>sex: 49% female<br>ethnic group: 100% Black	✓	✓	Higher family social capital and lower levels of neighbourhood danger were associated better outcomes. Higher levels of community social capital promote higher family social capital.
Aim: To assess how social capital, neighbourhood dangerousness and positive parenting relate to psychosocial adjustment (including internalising & externalising behaviours).					

Dufur et al 2008<br>USA<br>high quality	cross-sectional survey (National Longitudinal Survey of Youth)<br>data collected 1994	size: 1,833<br>age group: children & adolescents<br>age range: 5-14 yrs<br>sex: not stated<br>ethnic group: not stated	✓	✓	Higher levels of family social capital were protective in context of general mental health/behavioural problems. No role for school-based social capital.
Aim: To explore the role of family and school social capital in the promotion of positive socialization (including over and under-controlled behaviours).					
Feldman et al 2010<br>Israel<br>high quality	longitudinal cohort study<br>children followed from 3 months to 13 yrs	size: 31<br>age group: preschool, children & adolescents<br>age range: 3 months-13 yrs<br>sex: 48% female<br>ethnic group: not stated	✓	×	Across childhood, parent-child relationships high in positive, and low in negative characteristics were predictive of better adjustment in adolescence.
Aim: To observe mother-child interactive behaviours and to test associations with internalising and externalising behaviours in adolescence.					
Galboda-Liyanage et al 2003<br>UK<br>high quality	cross-sectional survey	size: 559<br>age group: preschool<br>age range: 3.5 yrs<br>sex: 49% female<br>ethnic group: not stated	✓	×	Higher quality parent-child relationships were associated with lower levels of behavioural problems.
Aim: To test the hypothesis that a low level of mother-school child joint activity is associated with behaviour problems.					

Harpham et al 2006<br>Vietnam<br>high quality	cross-sectional survey	size: 2,907<br>age group: preschool & children<br>age range (preschool): 6-18 months<br>age range (children) 7.5-8.5 yrs)<br>sex: not stated<br>ethnic group: not stated	×	✓	Maternal social support, civic engagement and a composite measure of community social capital associated with poorer mental health and increased behavioural problems in children. No role for group membership.
Aim: To explore the association between maternal social capital and child physical and mental health**.**					
Maynard et al 2010<br>UK<br>moderate quality	cross-sectional survey<br>data collected 2003	size: 4,349<br>age group: adolescents<br>age range: 11-13 yrs<br>sex: not stated<br>ethnic group: 28% White	✓	×	Positive parent-adolescent relationships associated with better mental health and fewer behavioural problems. Higher levels of parental control associated with poorer mental health and increased behavioural problems.
Aim: To examine associations between perceived parental care control and psychological wellbeing.					
Maynard et al 2010<br>UK<br>moderate quality	cross-sectional survey<br>data collected 2003	size: 4,349<br>age group: adolescents<br>age range: 11-13 yrs<br>sex: 47% female<br>ethnic group: 28% White	✓	×	Adolescents from families with infrequent participation in joint activities reported increased poorer mental health and increased behavioural problems.
Aim: To explore whether family activities explain ethnic differences in mental health.					

Newman et al 2007<br>USA<br>moderate quality	cross-sectional survey (National Survey of America's Families)<br>data collected 1999	size: 11,809<br>age group: adolescents<br>age range: 12-17 yrs<br>sex: 49% female<br>ethnic group: 67% White	✗	✓	Increased adolescent social support networks and parental attendance at religious services predictive of better outcomes. Knowledge of community resources inconsistent, with some protective and some risk associations. No role for parental civic engagement.
Aim: To examine relations between adolescent and parental social integration, residential and school stability, family access to community resources and wellbeing (including internalising and externalising behaviours).					
Parcel et al 1993<br>USA<br>high quality	longitudinal cohort study (National Longitudinal Survey of Youth)<br>data collected 1986-88	size: 524<br>age group: children<br>age range: 6-8 yrs<br>sex: not stated<br>ethnic group: not stated	✓	✗	Children whose parent is in a complex occupation have fewer reported problems but mothers working only part-time was a risk. Fewer children in the house was predictive of better outcomes.
Aim: To explore the association between parental working conditions and family circumstances on children's social adjustment (over and under-controlled behaviour).					
Parcel et al 1994<br>USA<br>high quality	longitudinal cohort study (National Longitudinal Survey of Youth)<br>data collected 1986	size: 1,294<br>age group: preschool & children<br>age range: 3-4 yrs<br>sex: not stated<br>ethnic group: not stated	✓	✗	Having a father but not a mother working fewer than full time hours was a risk factor for behavioural problems.
Aim: To evaluate the impact of parental working conditions on social outcomes (including over and under-controlled behaviour).					

Parcel et al 2001<br>USA<br>high quality	longitudinal survey (National Longitudinal Survey of Youth)<br>data collected 1992-95	size: 1,833<br>age group: children & adolescents<br>age range: 5-15 yrs<br>sex: not stated<br>ethnic group: not stated	✓	✓	Being in a larger family is a risk but parental monitoring, more frequent attendance at religious services and attending a school with a higher quality environment were protective in relation to behavioural problems.
Aim: To investigate the effects of both family and school capital on social adjustment (including over and under-controlled behaviour).					
Slee et al 2007<br>Australia<br>high quality	cross-sectional survey	size: 500<br>age group: preschool & children<br>age range: birth-7 yrs<br>sex: not stated<br>ethnic group: not stated	✓	✓	No role for family or community social capital.
Aim: To examine the links between young children's wellbeing (including behavioural problems) and social capital.					
Wen et al 2008<br>USA<br>high quality	cross-sectional survey (National Survey of America's Families)	size: 20,667<br>age group: children & adolescents<br>age range: 6-17 yrs<br>sex: not stated<br>ethnic group: not stated	✓	✓	Being in a 2-parent family, less frequent parent-child arguments, being involved in extra-curricular activities and religious services were protective. No role for civic engagement.
Aim: To contribute to understandings of the links and pathways between family structure and child wellbeing (including emotional wellbeing and behavioural problems).					
