# Supplementary figures and images for: The association between social capital and mental health and behavioural problems in children and adolescents: an integrative systematic review
Source: BMC Psychol. 2014 Mar 26;2:7. doi: 10.1186/2050-7283-2-7 (PMC4270040; doi:10.1186/2050-7283-2-7)

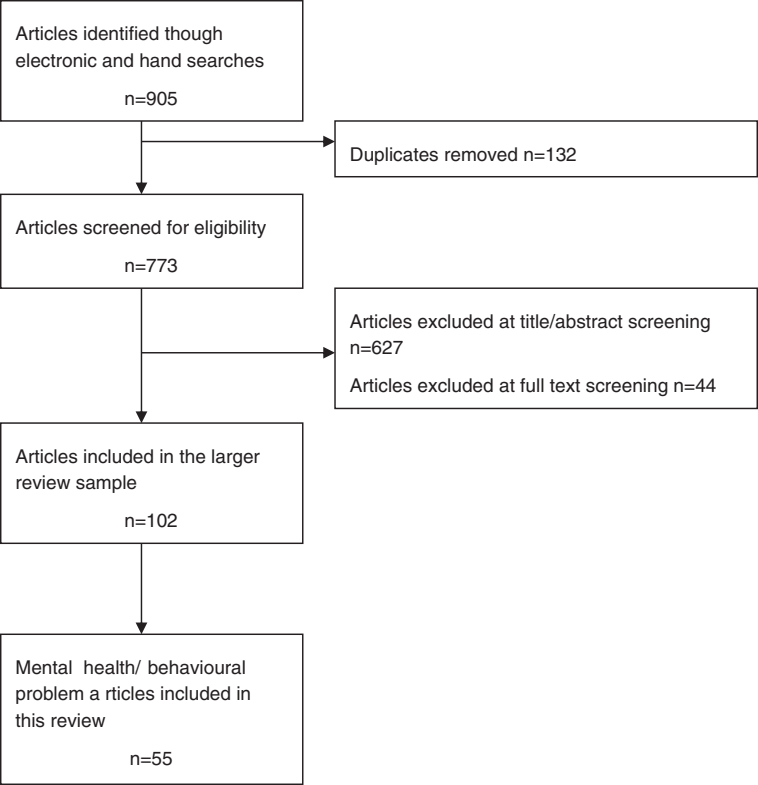

Supplement: Supplementary file 3 — Authors’ original file for figure 1 [file 40359_2013_9034_MOESM3_ESM.pdf]
